# Surveying hospital nurses to discover educational needs and preferences

**DOI:** 10.5195/jmla.2017.85

**Published:** 2017-07-01

**Authors:** J. Michael Lindsay, Sandy Oelschlegel, Martha Earl

## Abstract

**Objective:**

The author investigated the educational needs of nurses in an American Nurses Credentialing Center Magnet hospital to determine topics of interest, instruction time and delivery preferences, and interest in a research information skills certificate provided by the library.

**Methods:**

A 9-question survey was distributed to 1,500 nursing staff of the hospital through email.

**Results:**

Surveys were completed by 865 respondents, which represented a response rate of 58%. The majority of respondents were registered nurses, licensed practical nurses, and paramedics (81%), and day-shift workers (65%). For education topics, nursing staff placed the highest priority on finding health-related mobile apps for professionals and developing evidence-based research skills. For mode of delivery, respondents expressed a preference for unit-based in-service, computer-based tutorials, and hands-on computer training. Most (70%) respondents expressed an interest in participating in a research information skills certificate program.

**Conclusions:**

Our survey results reveal an avenue for reinvigorating and updating the library’s educational program to match the needs of nursing staff and may offer valuable insight for other libraries seeking to do the same.

## INTRODUCTION

The importance of medical libraries to nurses is well documented in the literature [1–4]. This is especially true for academic medical centers and hospitals that have achieved American Nurses Credentialing Center (ANCC) Magnet accreditation [3]. These settings have an increased expectation for nurses to research topics related to quality and safety of patient care as well as to do their own original research. Medical libraries traditionally offer instruction to assist nurses in becoming proficient in these information-related skills.

In addition, the concept of informatics in nursing has been more fully developed in the last two decades and now has a prominent role [5–7]. The American Nurses Association (ANA) defines nursing informatics as a “specialty that integrates nursing science, computer science and information science to manage and communicate data, information, knowledge and wisdom in nursing practice” [5]. The ANA nursing informatics competencies and other literature recognize different levels of nursing practice, with each level having a set of required competencies related to the collection, assessment, and application of knowledge-based information to evidence-based practice improvement [6, 8, 9]. In response to the ANA nursing informatics competencies, the Association of College and Research Libraries (ACRL) developed the Information Literacy Competency Standards for Nursing in 2013 to support nursing faculty and librarians who assist nursing students and nurses in developing information literacy skills for evidence-based nursing practice [7]. These standards guide and inform the development of education programs, both in nursing colleges and libraries that provide continuing education for the nursing workforce.

For nurses at the University of Tennessee Medical Center, the pursuit and maintenance of ANCC Magnet certification and the embrace of evidence-based practice has created a cultural change that is leading to increased research production and the need to develop skills in database searching, information literacy, presentation, and publication. In terms of nursing practice, as specified by ACRL’s Information Literacy Standards for Nursing, nurses must be able to “identify an information need, find and evaluate information, assess information, use information ethically and…use information for the purpose of best practice” [7]. Interactions with nursing staff convinced librarians at the Preston Medical Library that there was a need for education to support the ACRL standards at our institution. However, traditional face-to-face classes on information skills that librarians scheduled were sparsely attended.

The librarians realized that we needed a better understanding of nurses’ information needs, which led us to develop a needs assessment survey. In developing this survey, we solicited the help of several contacts with nursing education personnel that had previously been established through teaching library orientation classes for new staff, participating in the Nursing Professional Development Council, and serving on other nursing councils. We designed the survey with a number of goals in mind. Broadly speaking, we wished to determine what nurses needed to learn, when they wanted to learn, and how they wanted to learn. We also aimed to use the survey to gauge interest in the idea of combining library educational modules into the nurses’ existing e-learning system, which would both offer the ability to asynchronously earn continuing education (CE) contact hours and receive a research information skills certificate.

While developing the survey, librarians consulted with nurse educators regarding the concept of the research information skills certificate. All agreed that their existing e-learning system for delivering instruction to nurses, known as the “Talent Management System,” could accommodate library instruction modules designed to teach the skills outlined in the ACRL competencies and nursing informatics skills standards. We envisioned a program inspired by the Medical Library Association’s Consumer Health Information Specialization, which would include modules focused on knowledge of library services and resources, combined with basic searching of PubMed and CINAHL. Additional modules would focus on more advanced searching techniques and bibliographic management tools. If we uploaded the modules into the hospital’s Talent Management System, nurses would be able to use these materials from any location at any time and would have a permanent record of their work. As an additional incentive for nurses to use our program, the time they spent on these modules would be documented as CE contact hours, with which they could satisfy requirements for nursing certifications.

## METHODS

Working with nursing education staff, we developed a survey that included a base set of questions from previous internal surveys (i.e., job title, shift worked, and time of day that staff would prefer to attend a class) as well as questions to gauge interest in specific skills, times, modes of delivery, and interest in a research information skills certificate. The final survey was prepared using SurveyMonkey and consisted of nine questions. Three questions were multiple-choice, four were open-ended, and two questions utilized a variation of a Likert scale (supplemental appendix).

Prior to administering the survey, we sought and received approval from the hospital’s Nursing Research Council and received exemption from institutional review board (IRB) review. The survey was transmitted to the nursing email distribution list by the chief nursing officer’s executive assistant. We further promoted the survey through verbal announcements in nursing shared governance councils. The email containing the survey link was sent on a Monday morning to increase its chances of being read. Three weeks later, we sent a reminder email to maximize the response rate.

## RESULTS

We received 865 out of a possible 1,500 responses, for a response rate of 58%. Registered nurses, licensed practical nurses, and paramedics were the most frequent respondents (81%, n=702), followed by team leaders (7%, n=64); other (7%, n=60); nurse managers (3%, n=27); certified nursing assistants, nursing technologists, nurse anesthetists, and emergency department technologists (1%, n=7); social work/case managers (<1%, n=4); and a pharmacy technician (<1%, n=1). Not all questions were answered by all respondents.

**Figure 1:**
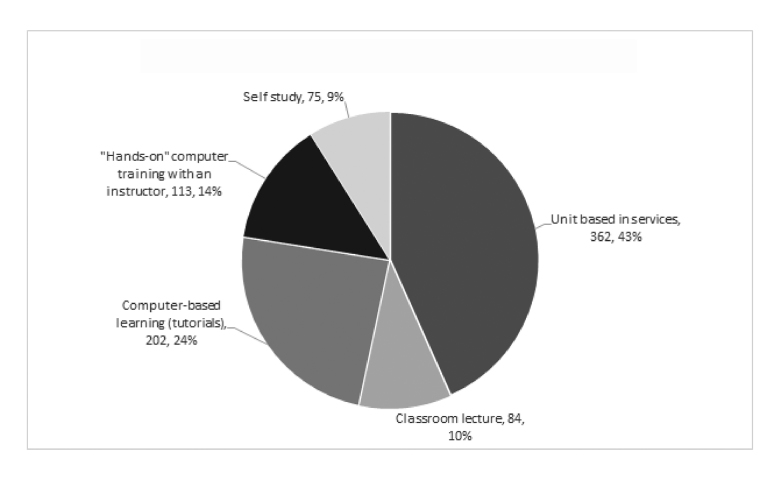
Preference for receiving education (first choice)

When asked about their preferred time of day to attend an educational offering, most respondents (75%, n=633) chose a time between 7:00 a.m. and 3:00 p.m. Overall preferences for receiving education were ranked in the following order: unit-based in-services, computer-based learning (tutorials), hands-on computer training with an instructor, classroom lecture, and self-study (Figure 1).

We expected differences between day-shift and night-shift staff in their preferences for when and how education would be received. At our institution, approximately 61% of nurses work days, which is reflected in our survey response distribution of more day-shift (65%, n=561) than night-shift (29%, n=254) staff. As only 24 respondents identified themselves as mid-shift staff, these respondents were not included in the analysis. Both day-shift (55%, n=234) and night-shift (51%, n=102) staff preferred unit-based instruction over the other options. The second most popular option for both day-shift (32%, n=126) and night-shift (38%, n=66) staff was computer-based learning (tutorials). The least popular option for day-shift staff was self-study (16%, n=38), whereas the least popular option for night-shift staff was classroom lecture (13%, n=15).

**Figure 2:**
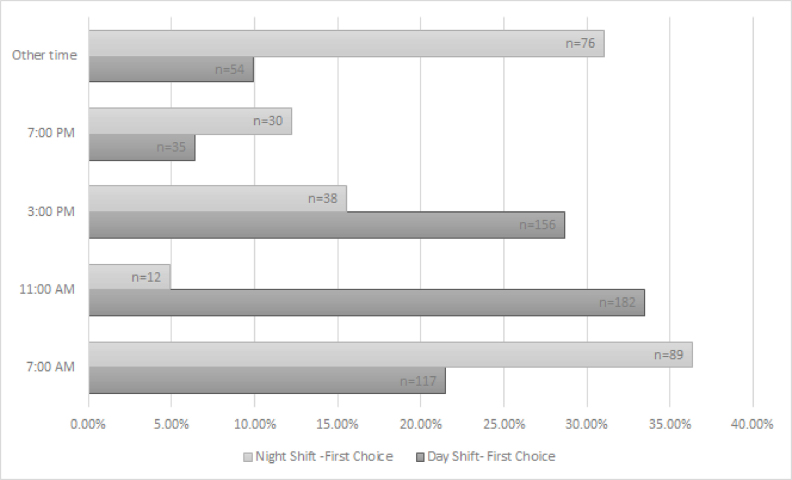
Preference for time of day to attend an educational offering (first choice)

There was also a distinction between day-shift and night-shift staff in their preference for the time of day of an educational offering. Day-shift staff clearly preferred taking classes during the middle of the day, with 11:00 a.m. being the most preferred time (Figure 2). By contrast, night-shift staff preferred a 7:00 a.m. start time for classes, which would roughly correspond to the end of their shifts. Also of note was the fact that “other time” was the second highest choice for night-shift workers, which could indicate a dissatisfaction with the choices presented.

We also found differences between night-shift and day-shift staff in their interest in receiving a research information skills certificate for taking classes from the library. Overall, 70% of respondents expressed interest in a certificate. Day-shift staff were significantly more likely to express interest in the certificate compared with night-shift staff (*p*<0.001, odds ratio 2.01, 95% confidence interval 1.44–2.79).

We also asked respondents to rank topics or skill areas, based on their value or interest. The top five most highly ranked topics were using health-related mobile apps for health professionals, developing evidence-based research skills (database search skills), helping patients find reliable health information on the Internet, using health-related mobile apps for patients, and using PubMed Clinical Queries to quickly find research. Of the fifteen possible topics, seven received an average rating of three or higher, indicating that respondents found these topics to be valuable (Table 1).

**Table 1:** Rankings of topics/skill areas (1=low value/interest, 5=high value/interest)

Answer options	Average rating
Using health-related mobile apps for health professionals	3.92
Developing evidence-based research skills (database search skills)	3.63
Helping patients find reliable health information on the Internet	3.56
Using health-related mobile apps for patients	3.40
Using PubMed Clinical Queries to quickly find research	3.31
Understanding the impact of low health literacy	3.17
Understanding how literature and the arts can help patients cope	3.04
Obtaining basic familiarity with library resources available at University of Tennessee Medical Center	2.99
Using PubMed to create new alerts and save searches	2.93
Using CINAHL searching techniques	2.83
Citing research effectively	2.82
Using literature and reflective writing in health care	2.79
Managing citations	2.66
Creating presentations and posters	2.61
Searching business databases	2.18

When asked to list three information skills that respondents would like to learn more about in the next year, they stated a desire to learn about research and publication, database searching, poster preparation, mobile apps for health care, and patient education. Practice-related skills were also mentioned. When asked how the library could assist with continued professional development, they indicated information on certification programs, how to obtain CE units, and help in accessing research and scholarly articles. Many respondents mentioned their plans to use the library more in the future as they pursue additional degrees. Finally, we invited respondents to provide any further comments or suggestions. One respondent suggested monthly information packets on new resources that are available; many respondents expressed their appreciation of the library and our services; and some wished there were more classroom space. However, some respondents stated that they were unaware of how to access library databases or how to request badge access to the library after hours.

## DISCUSSION

The challenge in implementing a new library educational program is to balance the desires of nursing patrons against librarian staffing and other realities. We discovered that the strongest preference for delivery of education was through unit-based in services, with librarians travelling to and providing instruction in units. However, day-shift staff have different preferences for the timing of educational events than night-shift staff do. Although we have traveled to and provided training in units in the past, these events were generally tied to short-term projects or the introduction of new resources or services. As a regular service offering from the library, classes outside of the library space and regular hours (e.g., 7:00 a.m.) could prove difficult to sustain.

Our survey results suggest other possibilities, however. The second most popular mode of education delivery was computer-based learning (tutorials). Preparing video tutorials that could be used anywhere, at any time, and that could be integrated into an existing online learning platform where nurses’ work would be documented presents many advantages and will be included in our future plans. In addition to computer-based learning, we found a desire for custom consultations and other classes. Although computer-based modules might appear to be the most efficient form of delivering instruction, respondents’ clear preference for unit-based instruction demonstrates the value of interpersonal interactions as the most effective means of teaching. Viewing these results comprehensively, we believe some combination of asynchronous classes, on-site training, and customized consultations should satisfy the need.

The responses to our survey provided us with confidence that we could design a research information skills curriculum and certificate that nurses and other hospital staff would value. As noted previously, while both day- and night-shift nurses expressed interest in a research skills certificate, we discovered that day-shift nurses were more interested than night-shift nurses were. Explaining this difference would require further investigation, but we believe that both day- and night-shift nurses would benefit from the certificate. While small classes provided to units and one-on-one sessions will continue to play a part in our overall education program, the research information skills curriculum would be delivered in an on-demand, asynchronous format to nurses and others.

The proposed research information skills curriculum would begin by teaching the basics in searching and information literacy and build up to more advanced searching and publishing topics. As mentioned previously, our plan is to provide a certificate inspired by the Medical Library Association’s Consumer Health Information Specialization. Similar to this program, our research information skills certificate would be a multilevel certificate program, beginning with a core set of modules focused on knowledge of library services and resources combined with basic searching of PubMed and CINAHL. To complete the first level of the certificate, participants would take additional courses from a list of electives, which would cover topics such as mobile apps for health professionals, MyNCBI, and health literacy. For the second level of the certificate, additional modules would be developed, focusing on more advanced searching techniques, bibliographic management tools, and reliable consumer health information on the Internet.

In previous research, both Dee and Rourke found that nurses appreciate the participation of librarians as partners in gaining nursing informatics skills that are needed in a Magnet environment [1–3], while Klem further defined the role of librarians in integrating information skills into nursing education [10]. There are several aspects to nursing informatics. Nurses desire guidance in how to apply research skills, a crucial part of nursing informatics, to clinical scenarios in an efficient way [10]. Simpson emphasized that skilled use of informatics technology, another aspect of nursing informatics, provides economic benefits in terms of the utilization of costly technologies and skilled workforce time while improving patient care [11]. Allen noted that evidence-based research and practice, which encompasses utilizing research skills to find knowledge-based information to apply to improving nursing practice, is essential for Magnet institutions [12]. Evidence-based research and practice requires that nurses have the necessary informatics skills to perform and integrate research into their work.

Nurses want to access the best evidence as quickly and effectively as possible, which is consistent with our survey results that the top five ranked skill or topic areas were health-related mobile apps for health professionals, evidence-based research skills (database search skills), use of PubMed Clinical Queries to quickly find research, help for patients to find reliable information on the Internet, and health-related mobile apps for patients [12]. Taken together, these different perspectives on nursing informatics align with Simpson’s finding that a patient-centered vision of what contemporary informatics skills encompass is important to nurses [11].

Although collaborating with the Nursing Professional Development and Nursing Administration Departments required additional time for library staff, this process resulted in greater visibility, greater credibility, and more investment from nursing for the project as a whole. For example, as a result of applying for IRB approval, we formally added nursing educational staff to the project, which brought it to the attention of staff who were preparing for ANCC Magnet recertification. Including nursing staff meant that our survey results could be presented in other venues, such as at Magnet and other nursing conferences. Another benefit of this collaboration was that even before the survey was prepared, we learned more about nursing CE, how nurses’ training was documented, and the priority that nursing leadership placed on those efforts. The nurse with whom we worked most closely suggested having the survey distributed by the chief nursing officer for the hospital, which most likely improved our response rate.

One limitation of our survey was that it was not mandatory; therefore, our results may be biased by reflecting the opinions of staff members who were more comfortable with technology. In addition, our response rate may have been lowered by factors such as delays in removing names from the email distribution list after staff members left the hospital or staff simply not reading their email or choosing not to participate. Because we chose to distribute the survey through the hospital’s nursing email distribution list and the primary participants were hospital nursing staff, the results might not be applicable to nurses in an academic setting, physicians, or other types of health care professionals. We also did not anticipate that so many respondents would indicate “other” as their job titles, which could suggest that they were clerical or environmental services workers or another type of nonclinical support staff. Furthermore, we did not consider any demographic data such as years of service or level of education, which could affect respondents’ existing level of information skills.

Our process of developing and implementing a needs assessment could interest medical librarians who support nursing informatics education. Based on our survey results, we conclude that nurses desire a research information skills certificate provided by the library. Our survey results are vital for reinvigorating and updating the library’s educational program to match the needs of users and can offer valuable insight for other libraries seeking to do the same.

## SUPPLEMENTAL FILE

Appendix Needs assessment survey questions
