# Supplementary material for: Surveying hospital nurses to discover educational needs and preferences
Source: J Med Libr Assoc. 2017 Jul 1;105(3):226–32. doi: 10.5195/jmla.2017.85 (PMC5490699; doi:10.5195/jmla.2017.85)
Supplement: Appendix [file jmla-17-226-a001.pdf]

JMLA Journal of the Medical Library Association

SC

Supplemental content to
J Med Libr Assoc. Jul;105(3): dx.doi.org/10.5195/jmla.2017.85
www.jmla.mlanet.org
© Lindsay, Oelschlegel, Earl 2017

Surveying hospital nurses to discover educational needs and preferences

J. Michael Lindsay, AHIP; Sandy Oelschlegel, AHIP; Martha Earl, AHIP

APPENDIX

Needs assessment survey questions

You are being asked to complete this survey as part of a research project being conducted by Michael Lindsay, Donna Patty, Sandra Oelschlegel, AHIP, and Martha Earl, AHIP, to determine continuing education needs at this institution. The survey is nine questions long and will take about five to ten minutes to complete. There are no known risks or personal benefits to completing this survey; however, the knowledge gained may benefit the Health Information Center and Preston Medical Library. Completing the survey is voluntary, and neither completing nor declining to complete the survey will affect your employment or standing at the University Health System or Graduate School of Medicine. The only identifiable data that will be collected are job title and shift if you choose to provide them. This information will only be used to determine education needs and time preferences.

1. What is your job title?
CNA, Nurse Tech, NA, ED Tech
Core Team/Radiology Transport
Health Unit Coordinator, ED Patient Rep, ENA
Medical Resident
Monitor Tech
Nurse Manager
Ortho Tech, Scrub Tech, Rad Tech
PCA
Pharmacist
Pharmacy Technician
Rehab Specialist (PT/OT/ST/AT)
Rehab Specialist Assistantt (PTA/OTA/STA)
RN, LPN, Paramedic
Social Work/Case Manager
Team Leader
Other

---

2. What shift do you typically work?
Days
Mid-shift
Nights
Other (please specify)

---

3. What time of day do you prefer to attend an educational offering?
7:00 a.m.
11:00 a.m.
3:00 p.m.
7:00 p.m.
Other (please specify)

JMLA Journal of the Medical Library Association

SC

Supplemental content to
J Med Libr Assoc. Jul;105(3): dx.doi.org/10.5195/jmla.2017.85
www.jmla.mlanet.org
© Lindsay, Oelschlegel, Earl 2017

4. How do you prefer to receive education? Please rank you top 3 preferences

	**First choice**	**Second choice**	**Third choice**
Unit based in services			
Classroom lecture			
Computer-based learning (tutorials)			
"Hands-on" computer training with an instructor			
Self study			

Other (please specify)

5. Are you interested in receiving a Health Information Skills certificate for taking classes from the library? *Please note that library classes would result in contact hours that could be applied to meet the requirements of other continuing education (CE) certificates and programs.*

Yes

No

6. Please rank the value of the following topics/skill areas on a scale of 1–5, with a 1 indicating that the topic is of low value or interest to you, and a 5 indicating that the topic is very valuable and of high interest to you.

	**1**	**2**	**3**	**4**	**5**
Evidence-based research skills (database search skills)					
Basic familiarity with library resources available at the University of Tennessee Medical Center					
CINAHL searching techniques					
Business database searching					
Presentation and poster creation					
Citing research effectively					
Managing citations					
Health-related mobile apps for patients					
Health-related mobile apps for health professionals					
Using PubMed Clinical Queries to quickly find research					
Using PubMed to create new alerts and save searches					
Helping patients find reliable health information on the Internet					
Understanding how literature and the arts can help patients cope					
Use of literature and reflective writing in health care					
Understanding the impact of low health literacy					

7. List three information skills you would like to learn more about in the next year:

8. How can the library assist you in your continued professional development?

9. Further comments or suggestions:
